# Psychological Traits and Social Factors Associated with Irritable Bowel Syndrome in Children

**DOI:** 10.3390/children13040521

**Published:** 2026-04-09

**Authors:** Daniela Pop, Ida Maria Lisa Aka, Radu Samuel Pop, Valentina Bota, Dorin Farcău

**Affiliations:** 1 Third Pediatric Discipline, “Iuliu Hațieganu” University of Medicine and Pharmacy, 400347 Cluj-Napoca, Romania; radu.samuel.pop@umfcluj.ro; 2 Third Pediatric Department, Clinical Emergency Hospital for Children, 2-4, Câmpeni Street, 400217 Cluj-Napoca, Romania; valentinabota@gmail.com (V.B.);; 3 Evangelisches Amalie Sieveking Krankenhaus, 22359 Hamburg, Germany; ida.aka@immanuelalbertinen.de; 4 Nursing Discipline, “Iuliu Hațieganu” University of Medicine and Pharmacy, 400347 Cluj-Napoca, Romania

**Keywords:** irritable bowel syndrome, anxiety, depression, children

## Abstract

Irritable bowel syndrome (IBS) and mental health disorders represent common and significant health concerns in pediatric populations. Objectives: This study aimed to evaluate psychological and social risk factors associated with IBS in children and to identify correlations with their gastrointestinal symptoms. Materials and Methods: Children aged 4 to 18 years diagnosed with IBS according to Rome IV criteria were eligible for inclusion. Both patients and parents completed a comprehensive questionnaire detailing gastrointestinal symptom characteristics. Additionally, all children underwent psychological assessment. Results: The study included 24 children with IBS, with a mean age of 12.7 ± 3.4 years. Anxiety was present in 54.2% of cases, and depression in 12.5%. Comparing children with IBS and anxiety to those without these, no statistically significant differences emerged regarding the duration and frequency of abdominal pain; however, abdominal pain intensity was significantly higher in children without anxiety (*p* = 0.04). The duration of IBS symptoms did not significantly differ in children with or without anxiety (*p* = 0.21). Impaired emotional self-regulation was identified in 54.2% of participants, and 41.6% exhibited vegetative symptoms in response to stress. Furthermore, 70.8% of parents and/or children reported experiencing a negative family event. Conclusions: The findings suggest that psychological characteristics and adverse family events are important risk factors associated with pediatric IBS. These factors should be systematically considered as integral components of clinical assessment and management.

## 1. Introduction

Irritable bowel syndrome (IBS) is an abdominal pain-related disorder of the gut–brain interaction [1]. The worldwide pooled prevalence in children is 8.8% [2]. IBS is poorly understood, and many factors are under investigation as possible causes, including genetic, infectious, psychological, antibiotic exposure, dysbiosis, allergies, visceral hypersensitivity, and motility disorders [3]. Due to the lack of a definitive etiology or pathophysiology, treatment options are symptom-focused and address certain risk factors. This uncertainty poses challenges for both families and clinicians, often leading parents and patients to question the diagnosis and seek alternative solutions, some of which are supported by limited evidence [4]. Given this complex landscape, it is important to consider associated psychological factors when managing pediatric IBS.

Mental health concerns are prevalent in children, as globally, 10% to 20% of children and adolescents are affected by mental health conditions [5]. The COVID-19 pandemic has both highlighted and worsened this issue [6]. The incidence of anxiety and depression is higher in children with chronic and life-limiting conditions [7]. These psychological disorders are frequently associated with functional gastrointestinal disorders, manifested by abdominal pain in children [8,9,10,11]. Anxiety disorders and IBS share common genetic pathways [12], such as impairment of the hypothalamic–pituitary–adrenal axis [13], serotonin (5-hydroxytryptamine-5-HT) level and signalling abnormalities [14], altered pain perception [15], and structural and functional changes in the brain [16]. It is not known whether psychological traits precede gastrointestinal symptoms or result from chronic, bothersome digestive complaints [17]. Both psychological and gastrointestinal symptoms can significantly impact quality of life, school adaptation, and relationships with family and peers [18]. Anxiety and depression are the most studied psychological risk factors linked to IBS in children [9]. However, other psychological traits or negative events during childhood might also interact with symptoms of IBS or their severity [19]. The study found that childhood emotional abuse and neglect were particularly significant psychological risk factors. Female sex, childhood emotional trauma, and negative events during adulthood were all independently associated with higher IBS prevalence [19]. As research highlights these associations, examining psychological risk factors in pediatric IBS warrants a focused study.

Beyond anxiety and depression, examining broader psychological personal resources is essential when addressing mental health in children with IBS. Factors such as resilience, coping strategies [20], emotional regulation [21], self-efficacy [22], and social support [23,24] may significantly influence how children perceive and manage gastrointestinal symptoms, potentially buffering stress and reducing symptom severity or frequency.

For example, children with stronger adaptive coping skills and higher resilience may better manage stressors that could otherwise worsen IBS symptoms [20,25]. Effective emotional regulation and supportive social environments can also reduce the negative effects of psychological distress on gut functioning [26,27,28,29]. Including psychological personal resources alongside risk factors gives a more complete understanding of the biopsychosocial mechanisms underlying IBS in children [30,31,32]. Reflecting this clinical complexity, the recent ESPGHAN/NASPGHAN guideline for treating IBS in children underscores the role of lifestyle changes, hypnotherapy, and cognitive behavioural therapy—interventions that target psychological aspects—in symptom management [3], further reinforcing the importance of psychological support as an integral part of care. Hypnotherapy has the strongest evidence [33,34,35] and is strongly recommended by the guideline [3]. Percutaneous Electrical Nerve Field Stimulation [36,37], amitriptyline [38,39] or cyproheptadine [40] may be suggested [3]. However, there is still a need for data to support therapeutic options in children with IBS and pediatric abdominal pain disorders in general [41].

The study aimed to assess the frequency of psychological risk factors among children with IBS and to examine how these factors relate to gastrointestinal symptoms. Additionally, possible risk factors such as family history of IBS or psychiatric disorders, low birth weight, and family events were evaluated.

## 2. Materials and Methods

The study included children aged 4 to 18 years old diagnosed with IBS according to Rome IV criteria. They were recruited between August 2024 and December 2025. The study was prospective, cross-sectional, and observational. Patients and their parents completed a questionnaire. This questionnaire detailed the characteristics of abdominal pain, such as localisation, intensity on a scale from 0 to 10 (numeric rating scale, from “no pain” to “the worst imaginable pain”), duration, and frequency. It also covered occurrences during the day or night, their relation to food, defecation, or stressful events. Additional information included stool pattern and other symptoms: vomiting, dysphagia, nausea, belching, early satiety, bloating, decreased appetite, sensation of incomplete defecation, urgency, involuntary stool loss, and rectal bleeding.

After appropriate medical evaluation and tailored investigations, the diagnosis of IBS was established. Diagnosis of IBS was based on the Rome IV criteria [1]. Patients with previously diagnosed neurological or psychiatric disorders were excluded from the study.

All children underwent psychological evaluation, and a report was prepared by a single psychologist, blinded to the IBS diagnosis. The psychological consultation included a clinical interview and the following assessments: cognitive tests (Raven [42] and Wechsler Intelligence Scale for Children (WISC) [43], Stanford–Binet Intelligence Scale [43]), developmental scales (Denver Developmental Screening Test [44] and Portage Assessment Scale [45]), emotional and personality tests (Achenbach System of Empirically Based Assessment (ASEBA) [46], Child Depression Inventory (CDI) [47], State-Trait Anxiety Inventory for Children (STAIC) [48]), and projective tests (House–Tree–Person, Family Drawing, Kinetic Family Drawing, Draw a Person Test).

Raven’s Coloured Progressive Matrices was used in children up to 6 years of age, and Raven’s Standard Progressive Matrices was used for ages 6 to 18 years as a nonverbal test to assess abstract reasoning and fluid intelligence through pattern completion tasks. WISC was used for children aged 6 to 16 years to evaluate verbal comprehension, working memory, processing speed, and perceptual reasoning. The Stanford–Binet Intelligence Scale was used to evaluate verbal, abstract, and quantitative reasoning, as well as short-term memory, in children under 6 years of age.

Denver and Portage developmental scales were used in children up to 6 years of age. The Denver Developmental Scale was used to assess language, fine motor, gross motor, and personal–social skills. Portage assessment scale was used to assess motor, socialization, language, self-help, and cognitive skills. Children with developmental delays were excluded from the study.

ASEBA evaluates emotional, social, behavioural, and thought problems. The ASEBA CBCL was completed by parents and administered to children aged 6–18 years. ASEBA YSR (self-report) was used in children aged 11–18 years. CDI, a self-report questionnaire, was used for children and adolescents aged 7 to 17 years to measure affective, cognitive, and behavioural signs of depression, like negative self-esteem, ineffectiveness, interpersonal problems, and negative mood. STAIC was used in children aged 8 to 14 years to assess state anxiety (situational anxiety, transient) and trait anxiety (stable anxiety tendencies).

House–Tree–Person, Family Drawing, Kinetic Family Drawing, and Draw a Person Test were used to evaluate anxiety, fear, emotional disturbance, mental disorders, family relationships, family functioning, and attachment representations in children aged 5 to 12 years. Projective techniques were used as qualitative tools within the clinical psychological evaluation. These were not quantitatively scored. Instead, findings were integrated with the clinical interview and behavioural observation.

The child’s emotional self-regulation was assessed during the clinical interview. Emotional regulation difficulties involve challenges in awareness of emotions, impulse control, and acceptance of emotions. In our reports, diagnosis relied on clinical observation and parental reports, especially when children showed intense emotional reactions during the assessment—such as tantrums or crying—that were disproportionate to the situation and difficult to calm, even with adult support. Increased impulsivity was observed: the child acted without thinking, hit, screamed, left abruptly, and struggled to wait or tolerate frustration. Emotional difficulties (e.g., anxiety, emotional dysregulation) were coded dichotomously (present/absent) based on convergence between projective indicators, clinical observation, and parental reports.

Due to the wide age range of participants (4–18 years) and the developmental variability within the sample, psychological assessment was tailored individually using age-appropriate, validated instruments. Consequently, not all participants completed the same tests, and the resulting variables are not directly comparable across the entire cohort. Instrument selection was tailored for each child or adolescent, aligning with age, developmental stage, and clinical traits. This method eliminated redundancies and delivered the most precise and relevant profile for each case. Perinatal risk factors (low birth weight, parents with IBS or mental illness) and family events (conflicts in the family, parents working abroad, moving, illness or death of a family member, etc.) were also included in the analysis.

Before participation, parents signed a consent form, and children provided assent. The study protocol received approval from the Ethics committee of the “Iuliu Hațieganu” University of Medicine and Pharmacy, Cluj-Napoca (AVIZ 139/19 July 2024).

Statistical analysis. Continuous variables were summarized as mean ± standard deviation (SD) when normally distributed and as median [interquartile range (IQR)] when not normally distributed. Categorical variables were expressed as percentages. The Mann–Whitney U test was used for comparisons between groups when data were non-normally distributed or sample sizes were small. Welch’s *t*-test was applied for normally distributed continuous variables with unequal variances after outlier assessment. Fisher’s exact test was used to compare categorical variables between groups. Effect sizes were calculated using Cohen’s d. A *p*-value < 0.05 was considered statistically significant. MedCalc version 22.023 was used for the statistical analysis.

## 3. Results

Twenty-four children diagnosed with IBS were included in the study. Demographic data and family medical history of the patients are summarized in Table 1.

Data regarding symptoms and signs of children with IBS are detailed in Table 2.

At least one adverse family event was reported by 17/24 (70.8%) patients and parents. The patients’ psychological traits and types of family events found in our study are summarized in Table 3.

Children with IBS and anxiety were compared with those without anxiety. The results of this comparison are summarized in Table 4.

The duration of IBS symptoms did not significantly differ between children with anxiety (median = 6 months, IQR = 3–12) and those without anxiety (median = 3.5 months, IQR = 3–8) (Mann–Whitney U = 41, *p* = 0.24). After removal of extreme outliers, the mean duration was 8.7 ± 6.3 months in the anxiety group and 5.9 ± 3.6 months in the non-anxiety group. The difference was not statistically significant (Welch’s t = 1.30, *p* = 0.21), although a moderate effect size was observed (Cohen’s d = 0.56).

Abdominal pain intensity was significantly higher in the group without anxiety. Abdominal pain intensity did not differ significantly in those with good emotional self-regulation (median 6 months, IQR 6–8) as opposed to those with impaired emotional self-regulation (median 7 months, IQR = 6–8) (*p* = 0.63). There was no statistically significant difference in the intensity of abdominal pain in those with conflicts in the family or those without (*p* = 0.32).

Abdominal pain was present both during the day and night in 11/24 (45.8%) patients, while 13/24 (54.2%) reported abdominal pain only during the day. Patients who had anxiety were 2.26 times (relative risk) more likely to have abdominal pain during the night compared to those without anxiety, and the odds ratio was 4.3, but the results did not reach statistical significance.

Anxiety was associated in 7/9 (77.8%) patients with IBS predominantly manifested with constipation, 5/11 (45.5%) children with IBS predominantly manifested with diarrhea, and 1/4 (25%) patients with a mixed form of IBS. The association of anxiety was strongest with the form of IBS manifested predominantly with constipation, but without statistical significance (*p* = 0.12).

There was no statistically significant difference between the children with impaired emotional self-regulation and those without this psychological trait regarding the age, duration, or frequency of the abdominal pain (*p* > 0.05).

## 4. Discussion

This study assessed psychological characteristics and family adverse events in children with IBS. Most of the patients were girls (70.8%). IBS predominantly manifested with diarrhea was the most frequent subtype, closely followed by the constipation predominant subtype. There were no patients with an unclassified subtype.

Over 70% of patients and/or their parents reported at least one adverse family event during psychological consultations, with some noting multiple events. Almost half of the children in our study came from families with conflicting events, and almost one-third had a family member who died or was ill. Patterns revealed significant family disruptions in recent decades, including parental migration, which sometimes left children behind or led them to move. Around 16% of the children with IBS had at least one parent working abroad. This study strengthens the idea that family stress and instability are highly prevalent in pediatric IBS, even if causality remains debated.

The relation between adverse childhood events like abuse, neglect, or other potentially traumatic events has been reported by several studies, but conclusions are inconsistent, and results are biassed by the quality of the studies or design [49]. Both adverse childhood events and adverse adulthood experiences are associated with a higher risk of IBS in adults [32]. The long-term impact of these events in childhood is supported by studies in adult patients with IBS, which are correlated with a higher risk, particularly in women, and describe a distinct phenotype of IBS [50,51,52].

Fu et al. found anxiety in 21% of the children with IBS, and depression in 14% of them [53]. More than half of the children diagnosed with IBS in our study exhibited anxiety, and 12.5% depression. The most frequent form of anxiety was performance anxiety. When the intensity of abdominal pain (measured on a numerical scale from 0 to 10) was compared in children with and without anxiety, there was a statistically significant difference, with a more intense abdominal pain reported in the group of children without anxiety. This contradicts previous findings in which the severity of abdominal pain in children was correlated with anxiety [25,54,55]. Only Hollier et al. published results in children with IBS; the rest of the studies included other gut–brain interaction disorders as well [25]. However, Hollier et al. use a different numerical scale to evaluate pain intensity, not a numerical 0-to-10 rating scale. Anxiety and depression can alter signalling between the central nervous system and the enteric nervous system. This can lead to increased visceral sensitivity (heightened perception of gut pain) and altered gut motility (constipation or diarrhea patterns). The study found higher abdominal pain intensity in children without anxiety. It might be explained by the fact that children with anxiety may have chronic hypervigilance, leading to earlier reporting of symptoms, adaptation, or altered perception over time. The limited sample size may have influenced our results.

Higher scores for anxiety and depression were correlated with the duration of symptoms in previous studies [56]. We did not find statistically significant differences between children with IBS with or without anxiety regarding the duration of their symptoms. Non-anxious children might report pain only when it becomes more severe. Chronic stress activates the hypothalamic–pituitary–adrenal axis. This leads to elevated cortisol levels, alters gut permeability and inflammation, and affects motility and microbiota composition. This could contribute to a longer symptom duration and persistent IBS symptoms in anxious or depressed children. Somatisation and pain catastrophising mediate the relationship between IBS symptoms and anxiety, and they should be treatment targets [25]. Vegetative manifestations (autonomic symptoms) such as palpitations, dizziness, sweating, or flushing were reported by nearly 42% of our patients in relation to stressful events. Ruška et al. found symptomatic autonomic dysfunction in 61.5% of the children with IBS [57]. Health-related quality of life could be improved by treating somatisation and functional disability in children with IBS [58]. These are important treatment targets in cognitive behavioural therapy and stress regulation.

Emotional instability and less involvement in social activities were correlated with IBS in children [59]. Emotional instability modulates the perception of visceral stimuli, correlating with lower pain thresholds and higher pain scores [59]. Impaired emotional self-regulation was diagnosed in more than half of the children with IBS in this study. Difficulty connecting emotionally with others was found in almost 38% of the patients with IBS. Pandemic-related social disruption has been recognized as a factor with a negative impact on children with gastrointestinal disorders, including IBS [24].

High rates of school absenteeism have been found in children with IBS, both in primary care settings and in hospitalized children with IBS [60]. Anxiety, gastrointestinal symptoms, and somatisation have a negative impact on school performance [59,61,62]. Almost 38% of our patients admitted to having difficulties in adapting to school or kindergarten. Robbertz et al. recently showed a positive correlation between gastrointestinal symptoms and anxiety during the COVID-19 pandemic, with online schooling being associated with worsening of the symptoms [63].

A recent review identified low birth weight and parental IBS or mental illness as risk factors for IBS in children [31]; however, our group did not include children with birth weight under 2500 g, and few reported parental IBS or mental illness.

The study is a prospective one, with data on gastrointestinal symptom characteristics collected uniformly using a questionnaire based on Rome IV criteria. The analysis included only patients diagnosed with IBS and excluded individuals with other gut–brain interaction disorders. Patient selection was based on the specific diagnosis of IBS to enhance the specificity of the findings in this group compared with most previous studies.

The psychological assessment did not rely solely on standardized quantitative methods. Our integrative framework combined quantitative tests and qualitative approaches—clinical observation, projective techniques, and contextual analysis—to produce a comprehensive, nuanced understanding of participants’ psychological functioning. The study has some limitations that must be acknowledged. Patients were recruited from only one centre. The study group was small, and a control group was not included in the analysis. It is a cross-sectional study. Children had a wide age range, and during psychological consultation, different scales were used for cognitive, developmental, personality, and emotional assessment. This heterogeneity limits direct comparability across the full cohort. Further studies on our patients aim to use the same scales to evaluate psychological factors, suitable for different age groups, with a uniform evaluation of specific variables, and to include more patients over a period of 5 to 10 years to ensure statistical significance of the results.

In considering psychosocial factors, a high proportion of participants reported adverse family experiences, including conflict, illness, death, and parental migration. This supports existing but inconsistent evidence linking early life stressors to IBS risk, notably described in adult populations. Rates of anxiety were notably higher than previously reported, while depression prevalence was comparable. However, in contrast to most prior studies, greater abdominal pain intensity was observed in children without anxiety, and no association was found between psychological symptoms and illness duration. A substantial proportion of patients exhibited somatisation, autonomic symptoms, impaired emotional regulation, and social difficulties, which aligns with literature highlighting that psychosocial factors modulate symptom perception and functional impairment, including school difficulties. While established risk factors such as low birth weight and parental IBS were not identified, possibly due to sample limitations, these findings reinforce the biopsychosocial model of pediatric IBS and contribute new insights into the role of family disruption and emotional functioning.

## 5. Conclusions

Psychological traits and family events are important risk factors for IBS in children and should not be overlooked in clinical management. Specifically, data suggest that adverse experiences—such as the illness or death of a family member and intrafamilial conflicts—are associated with a significant proportion of pediatric IBS cases. These factors can elevate stress levels, negatively influence emotional regulation, and thereby favour the expression of psychological distress through somatic symptoms.

In this regard, family relationships and contextual factors must be integrated into both assessment and therapeutic intervention. An exclusively somatic approach is insufficient; a biopsychosocial perspective is necessary to optimize clinical outcomes and the well-being of children with IBS.

## Figures and Tables

**Table 1:** Demographic data of the patients with IBS.

Number of patients	24
Age (years) mean ± SD range	12.7 ± 3.4 5–17 years 6 months
Gender Girls Boys	17/24 (70.8%) 7/24 (29.2%)
Rank of the child first second third eight	12/24 (50%) 10/24 (41.7%) 1/24 (4.2%) 1/24 (4.2%)
Low birth weight (under 2500 g)	0/24 (0%)
Family history of IBS	2/24 (8.3%)
Family history of psychiatric disorders	1/24 (4.2%)

IBS—irritable bowel syndrome.

**Table 2:** Symptoms, signs, and IBS subtype.

Duration of symptoms until evaluated by a pediatric gastroenterologist	6 months (median)
Intensity of abdominal pain	6.5 (median)
Frequency of abdominal pain daily once/week 2 times/week 3 times/week	17/24 (70.8%) 2/24 (8.3%) 2/24 (8.3%) 3/24 (12.5%)
Duration of abdominal pain less than 1 h 1–2 h 2–4 h continuous	13/24 (54.2%) 6/24 (25%) 2/24 (8.3%) 3/24 (12.5%)
Bloating	14/24 (58.3%)
IBS subtype diarrhea constipation mixed	11/24 (45.8%) 9/24 (37.5%) 4/24 (16.7%)

IBS—irritable bowel syndrome.

**Table 3:** Psychological and social factors associated with IBS in children.

Anxiety Performance anxiety Social anxiety Generalized anxiety	13/24 (54.2%) 6/13 2/13 5/13
Depression	3/24 (12.5%)
Panic attacks	3/24 (12.5%)
Impaired emotional self-regulation	13/24 (54.2%)
Difficulty concentrating	5/24 (20.8%)
Difficulty connecting emotionally with others	9/24 (37.5%)
Vegetative manifestations in stressful situations	10/24 (41.6%)
Distrust of one’s own strength	6/24 (25%)
Body image dissatisfaction	2/24 (8.3%)
Sibling rivalry	5/24 (20.8%)
Difficulties in adapting to school/kindergarten	9/24 (37.5%)
Sleep disturbances	2/24 (8.3%)
Family events One or both parents working and living abroad Illness or death of a family member Moving Conflicts in the family Other potentially traumatizing family events	4/24 (16.6%) 7/24 (29.1%) 3/24 (12.5%) 11/24 (45.8%) 6/24 (25%)

**Table 4:** Comparison of IBS symptoms in children with and without anxiety.

	With Anxiety	Without Anxiety	*p*
Number of patients with irritable bowel syndrome	n = 13	n = 11	
Intensity of pain mean ± SD median range IQR	6.23 ± 0.93 6 5–8 6–7	7.36 ± 2.29 8 3–10 5–9	0.04
Duration of abdominal pain less than 1 h more than 1 h	6/13 (46.2%) 7/13 (53.8%)	7/11 (63.3%) 4/11 (36.4%)	0.41
Frequency of pain daily once, twice, or three times/week	9/13 (69.2%) 4/13 (30.8%)	8/11 (72.2%) 3/11 (30.8%)	1
Pain during the day and night Pain only during the day	8/13 (61.5%) 5/13 (38.5%)	3/11 (27.3%) 8/11 (72.7%)	0.11
IBS subtype Diarrhea Constipation Mixed	5/13 (38.5%) 7/13 (53.8%) 1/13 (7.7%)	6/11 (54.5%) 2/11 (18.2%) 3/11 (27.3%)	0.12

SD—standard deviation, IQR—interquartile range.

## Data Availability

The original contributions presented in the study are included in the article, further inquiries can be directed to the corresponding author.

## Abbreviations

The following abbreviations are used in this manuscript:

IBS	irritable bowel syndrome
IQR	interquartile range
SD	standard deviation
